# Therapeutic approach to patients with rheumatoid arthritis and chronic HBV/HCV infection

**DOI:** 10.1186/1471-2334-14-S7-P73

**Published:** 2014-10-15

**Authors:** Sînziana Daia-Iliescu, Bogdan Toia, Daniela Opriş, Ruxandra Ionescu

**Affiliations:** 1 “Sf. Maria” Clinical Hospital, Bucharest, Romania; 2 Carol Davila University of Medicine and Pharmacy, Bucharest, Romania

## Background

Treating chronic HCV/HBV-infected patients with concomitant rheumatoid arthritis (RA) may be a challenge to the clinician. The liver disease limits to some degree the treatment for the rheumatic disease as the drugs used are hepatotoxic or at risk of infection reactivation. The aim of the study is to evaluate the particularities and the safety of RA treatment in patients with both conditions and to investigate the prevalence of HBV/ HCV-infections in RA patients.

## Methods

We performed a cross-sectional analysis of all HCV/HBV or co-infected patients with concomitant RA admitted between 2009-2014, assessing the clinical, laboratory, treatment data and verifying the statistic correlations.

## Results

The study included 66 patients, with both chronic liver infection and RA, of which 35 (53%) patients had HCV, 24 (36.3%) had HBV and 7 (10.6%) were co-infected. The median age was 61±11 years, sex ratio male/female 13/53. The HCV-infected patients had a very active rheumatic disease activity score (DAS28) (5.03±1.43) while the HBV and co-infected patients had a moderate DAS (DAS28=4.06±1.68), respectively DAS28 (3.05±1.74) (p=0.015). Therapeutic options include disease-modifying anti-rheumatic drugs (DMARDs), classic or biologic as well as corticosteroids (CS). In our study, most patients, 25 (37.8%) received hydroxychloroquine, 9 (13.6%) patients received methotrexate and 7 (10.6%) patients sulphasalazine alone. Combinations included hydroxychloroquine + sulphasalazine in 5 (7.57%) patients and methotrexate + hydroxychloroquine in 4 (6%) patients.

In 1 (14.2%) patient out of 7 receiving biologic treatment 6 (85%) infliximab, 1 (14, 2%) etanercept, 5 (71, 42%) rituximab – current or prior treatment, reactivation of the HBV infection occurred, during the 6^th^ cycle of therapy.

The rise in the liver enzymes, leading to discontinuation were seen in 14 (56%) patients receiving methotrexate, followed by 7 (10.6%) patients receiving sulfasalazine, and 1 (4.3%) in the hydroxychloroquine group. Significantly more patients in the HCV group 27 (80%), compared with the HBV group 24 (45.8%), had an active disease and were given low dose corticosteroids (p=0.028).

## Conclusion

The presence of the liver infection limits to some degree the therapy for RA. Classic DMARDs such as methotrexate should be closely monitored as they proved most hepatotoxic and led to frequent discontinuation. Hydroxychloroquine was best tolerated. HCV-patients with RA often require more aggressive therapy, including biologics, which also have to be used with caution. The prevalence of HCV infection was higher than HBV infection in the study RA group. Also HCV-infected patients had a more active rheumatic disease and used corticosteroids more frequently.

